# What Does the Shape Space of T Cell Epitopes Look Like?

**DOI:** 10.1111/imr.70152

**Published:** 2026-08-07

**Authors:** Franka Buytenhuijs, Gijs Schröder, Johannes Textor

**Affiliations:** ^1^ Data Science, Institute for Computing and Information Sciences Radboud University Nijmegen the Netherlands; ^2^ Medical Biosciences Radboudumc Nijmegen the Netherlands

## Abstract

*Shape space* is a decades‐old conceptual model of antibody–antigen interactions that underlies antigenic maps used to trace viral evolution. Here, we apply this concept to T cell receptors (TCRs) and the peptide–MHC complexes (pMHCs) that they recognize. We start by reviewing the history of shape space and its deep connections to concepts in statistical physics (energy landscapes) and computer science (complexity theory). Leveraging these connections, we propose a model in which TCR‐pMHC binding relies on multiple, possibly conflicting, structural constraints—implying that pMHCs recognized by the same TCR occupy several disjoint, non‐convex regions in shape space. Our model makes two central predictions: (1) even small pMHC structure alterations can have major effects on immunogenicity; (2) two pMHCs recognized by the same TCR can have very different structures. We show that published TCR‐pMHC interaction data generated by mutagenesis assays lend some support for this idea. We conclude by discussing implications of a multispecific and non‐convex T cell epitope shape space for the prediction of immune responses in cancer immunotherapy and other applications.

## 1 Shape Spaces and Immunogenicity Landscapes

T cells are key effector cells in the immune system that react to “anomalous” peptides presented on MHC class I or class II molecules. Such peptides are typically 9–14 amino acids long, which can store an amount of information that is comparable to a short English word. The T cell population relies on this information to “decide” whether to mount an immune response. This *self‐nonself discrimination* must be done swiftly to deal with invading pathogens, but also reliably to prevent immune responses against the body's own cells [1, 2].

In a 1979 landmark study, Perelson and Oster studied self‐nonself discrimination for B cell epitopes [3]. They asked how many different antibodies are required to fully cover the entire possible universe of potential foreign antigens, while at the same time reliably discriminating them from self? Central to their analysis was the concept of *shape space*, which they defined as an abstract space representing possible molecule shapes in which both antibodies and antigens live. Antigens that are close in shape space would be recognized by the same or similar antibodies. Shape space was not merely a theoretical concept—several attempts were made, for example, to deduce its dimension from either the sizes of B cell repertoires [3, 4] or from *panel data* in which the immunogenicity of different virus strains is evaluated against different antisera [5, 6, 7]. Both approaches arrive at a low dimensionality of around 5–10, although these estimates are debatable and the real dimension could be much higher [8].

**FIGURE 1:**
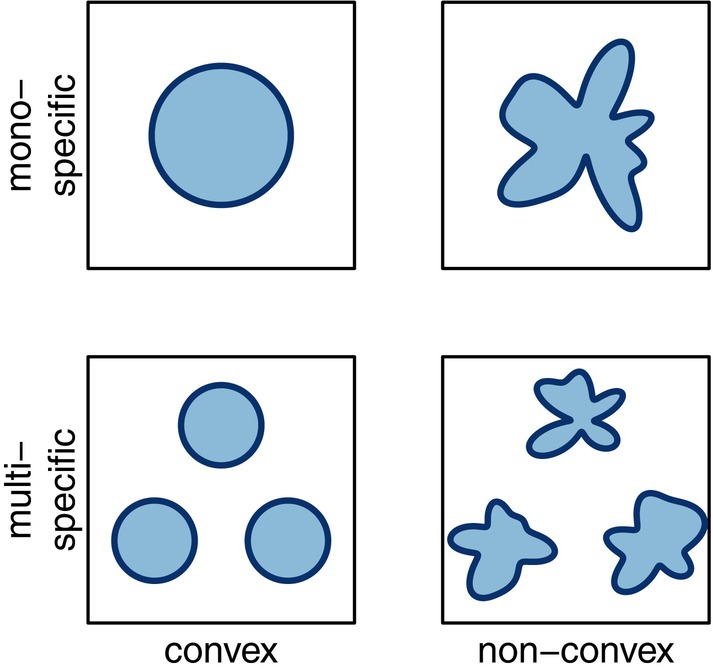
Recognition regions in shape space. Each point in shape space represents one antigen, and the colored areas represent antigens recognized by the same antibody or TCR (recognition regions). These regions can consist of one (top) or multiple (bottom) sub‐regions, which can be convex (left) or rugged (right).

We can define the *recognition region* (Figure 1) of a given antibody as the part of shape space that contains the antigens it recognizes—which we take here to mean that the binding affinity between these molecules exceeds a set threshold. In a simple case, the recognition region is shaped like a ball or hypersphere, and it contains a unique center point representing the best recognized antigen, called the *shape complement* [6] or *binding motif* [2]. If the recognition region forms a single, connected patch, we call it *monospecific* (note that this still allows TCRs to recognize multiple similar pMHCs). Perelson and Oster also considered *multispecific* recognition, in which the recognition region is a union of disconnected components. In this paper, we additionally distinguish between *convex* recognition regions (such as a ball) and *non‐convex* ones for which there is no perfect relationship between distance to a center antigen and immunogenicity.

Recognition regions are closely related to *energy landscapes* in theoretical physics [9, 10]. Energy landscapes have been used in biophysics to explain why certain amino acid chains can fold into stable structures [11]. The basic idea is that we consider a system that can be in a large number of different states (such as the set of possible spatial configurations of an amino acid chain), with an energy function assigned to each state. Together with the possible transitions between the states, this creates an energy landscape that defines the stability of different configurations in the system as well as the time it takes to transition between them. Stable states correspond to deep local minima in the energy landscape. A landscape with many local minima separated by intermediate states of higher energy is called *glassy*, whereas a landscape with a single, deep global minimum that can be easily reached from most other states is called a *funnel*. The rugged structure of glassy energy landscapes is generated by *frustration*: the need to satisfy multiple, potentially conflicting constraints. For example, free energy in a protein structure can be reduced by establishing bonds between some of its amino acids; however, establishing one bond could make it harder or impossible for some other bonds to form.

Here we argue that a recognition region in shape space is essentially an energy landscape: it is the set of all antigens whose free binding energy to the considered antibody is lower than a set threshold. We will refer to this energy landscape that underlies the recognition region of a given antibody or TCR as its *immunogenicity landscape*—specifically, recognition regions are areas around local maxima in this landscape. Rather than spatial configurations of a fixed protein, the states in immunogenicity landscapes are different antigens, and the transition function turns one antigen into another—for example, by swapping out an amino acid. In this view, a *convex* recognition region would correspond to a *smooth* immunogenicity landscape where the binding energy changes gradually and predictably as changes to the antigen are made. For non‐smooth immunogenicity landscapes, recognition regions could be non‐convex, multispecific, or both.

## 2 The TCR‐pMHC Immunogenicity Landscape

**FIGURE 2:**
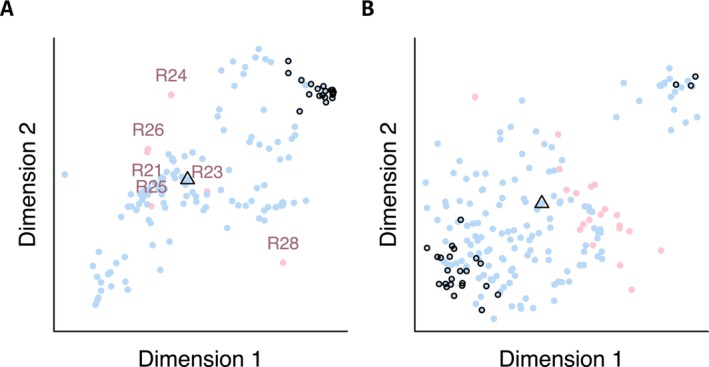
Two‐dimensional embeddings of the T cell epitope shape space generated from data by Drost et al. [14]. Each plot represents T cell receptors (red) and peptides (blue) as part of the same space. Every peptide is a single‐point mutation of a source peptide (marked with a triangle) recognized by all TCRs. Peptides marked with a black circle are no longer recognized by any of the TCRs; every other peptide is recognized by at least one TCR. (A) Six TCRs that recognize the human cancer neo‐epitope VPSVWRSSL evaluated against 133 point mutations. (B) Twenty TCRs that recognize the common HLA‐A02:01‐restricted epitope NLVPMVATV evaluated against 172 point mutations.

Let us illustrate these concepts by constructing a shape space map for T cells using a method originally developed for antibodies: explicit shape space mapping using *panel data* [6, 7]. In antibody panel data, routinely collected by the WHO for influenza monitoring, one tests the ability of different antisera to block the agglutination of red blood cells by different influenza virus strains [7]. The resulting data could be seen as a partially observed distance matrix—the lower the inhibiting capacity, the larger the distance—where distances are only observed between antigens and antisera, but not within antigens or within antisera. One can then use *multidimensional unfolding*, a version of multidimensional scaling that allows for missing data [6, 12], to construct an embedding of this partially observed distance matrix in a Euclidean space (typically two‐dimensional). For T cells and their epitopes, we can generate the same kinds of shape space maps from TCR‐pMHC interaction data. Figure 2 shows such maps for TCR‐pMHC interaction data by Straub et al. [13] and Drost et al. [14], who measured the affinity of various TCRs to point mutations of a common pMHC epitope.

Constructing antigenic shape space maps by multidimensional unfolding assumes that the observed interaction strength matrix is generated by an underlying metric from which there is a monotone mapping to the observed interactions. In other words, we assume that the immunogenicity landscape is highly smooth. Depending on the exact version of unfolding, this may imply that recognition regions are connected (monospecificity, for geodesic metrics) or convex (Euclidean metrics). If these assumptions hold, then we can predict the immunogenicity of a new antigen by mapping it onto the shape space and asking how close it is to an already known immunogenic one. Similarly, we could estimate the amount of existing immune memory in a population by measuring the distance of a new antigen to previously circulating ones—exactly what *antigenic maps* are designed to do [7, 15, 16].

From Figure 2, it appears that the underlying TCR‐pMHC immunogenicity landscape may not be smooth, or two dimensions may be insufficient to represent it. There is, for example, no perfect separation between the pMHCs not recognized by any TCR (black circles) and the other pMHCs (blue circles). On the other hand, the map does reveal some interesting substructure, such as an “outlier” TCR (R28) in Figure 2A and an apparent cluster of pMHCs in Figure 2B, which could represent a different binding mode. The idea that such structure might hold useful information even if the method's assumptions are not fully met underlies the use of such antigenic maps for B cells. But can we argue from first principles whether or not we can expect these assumptions to hold for TCR‐pMHC interactions?

## 3 Classic Receptor‐Antigen Interaction Models Imply a Smooth Immunogenicity Landscape

**FIGURE 3:**
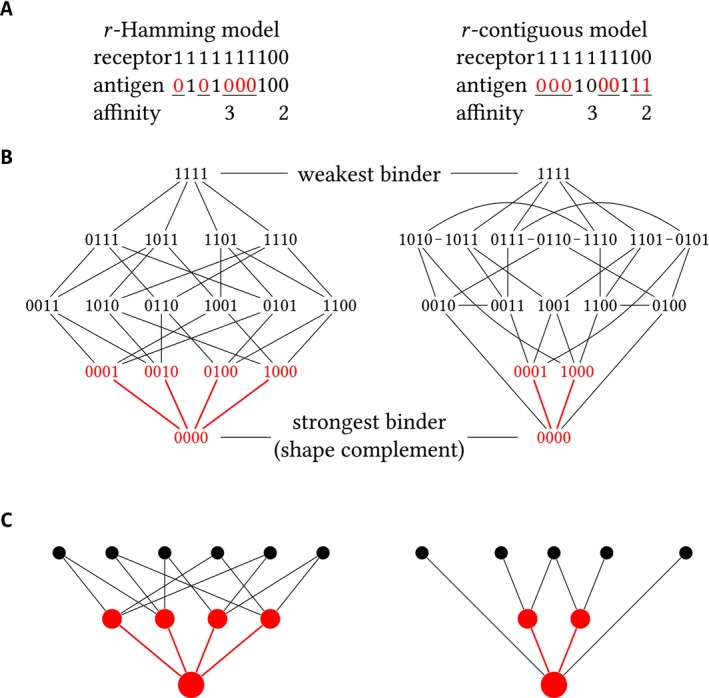
Common “string distance” models of receptor‐antigen interactions and their implied immunogenicity landscapes. (A) In a Hamming binding model, receptors and antigens are represented by strings, and the affinity between them is the number of complementary bits (underlined and highlighted). In the contiguous binding model, affinity is instead defined as the length of the longest contiguous stretch of complementary bits. (B) All strings of length 4 arranged according to their affinity to the receptor 1111 in each model. Lines connect strings that differ in at most 1 position. For Hamming shape space, every mutation decreases or increases affinity by 1. For contiguous shape space, there can also be a change of 2 (when middle positions are changed) or 0 (when border positions are changed). (C) Simplified representation of (B) focusing only on the most immunogenic epitopes (red) and their direct neighbors in (B). In both cases, this shows that immunogenic epitopes show a single, connected cluster.

Around the same time when shape space models were developed, computer scientists became interested in the human immune system as an inspiration for safe, reliable and adaptive computer algorithms, sparking a line of research now known as *artificial immune systems* [17, 18, 19, 20]. In a foundational paper, Farmer, Packard, and Perelson introduced the first shape space model based on *string* representations of receptors and antigens [21]. This type of model arises naturally from thinking of receptors and antigens as shapes and their complements—this is the computer science equivalent of the classical key‐and‐lock analogy often used in teaching to illustrate antigen–antibody binding [22]. In its simplest form, we model antigen and antibody as *strings* (taken here to be sequences of zeroes and ones), and define an *affinity function* (or *matching function*) between them. A common choice is the Hamming distance (Figure 3A, left), which counts the number of complementary bits between two strings [21, 23].

If we apply such a model to TCR‐pMHC interactions, what does it predict about the immunogenicity landscape? Modeling TCR‐pMHC interactions using the Hamming distance leads to convex, monospecific recognition (Figure 3B, left): Every point mutation on the TCR or pMHC side changes the affinity by exactly the same amount; there is a unique strongest pMHC for every TCR (obtained by changing all zeroes to ones and vice versa); and the ball of recognition (marked in red in Figure 3B) consists of several related pMHCs that are at most a fixed number of changes away from the shape complement.

Already in the 1980s, it was clear that the Hamming distance model of antigen–antibody interactions was overly simplistic, since it was known that not all mutations of an antigen affect immunogenicity equally. This motivated the development of the *contiguous distance* as an affinity function [24], which is a version of the Hamming distance where we require the complementary bits to occur in a contiguous stretch (Figure 3A, right). This induced a positional bias (Figure 3B, right): mutations in the middle of the string can reduce affinity by more than half, while mutations at the ends may not have any effect at all. This leads to recognition regions that are non‐convex (Figure 3B, right—some single‐bit mutants of the shape complement are inside the recognition region, others are outside). Still, the bitwise complement remains the unique strongest binder, and recognition remains monospecific: there is just a single region of connected binders. Therefore, we would still be able to predict an antigen's immunogenicity from nearby, similar antigens once we consider the positional bias in the effect of mutations. Overall, despite the more complex behavior of the contiguous distance function, the generated immunogenicity landscape remains qualitatively similar to the Hamming landscape (Figure 3C).

Thus, early models of TCR‐pMHC interactions based on strings with Hamming or contiguous distance functions led to smooth immunogenicity landscapes. This finding can be understood by thinking of immunogenicity in such shape spaces as the result of satisfying multiple constraints that are either independent (Hamming distance) or positively dependent (contiguous distance). From a machine learning point of view, this means that identifying and constructing the matching pMHC for each receptor should not be a very difficult task.

## 4 Multi‐Constraint Interaction Models Can Imply Smooth or Rugged Immunogenicity Landscapes

Do we expect the smooth immunogenicity landscapes implied by early shape models to actually hold for TCR‐pMHC interactions, or are these models missing something important? To address this question, we once again look to statistical physics for inspiration. There is a deep connection between the roughness of energy landscapes and the hardness (computational complexity) of certain problems in computer science [25, 26]. In these *constraint satisfaction problems*, solutions need to be found that satisfy multiple, possibly conflicting constraints. If we think of the number of unsatisfied constraints as a “free energy”, then solving such problems means finding minima in an energy landscape, and difficulty arises from the presence of many local, disconnected minima. It seems plausible to view TCR‐pMHC binding as a similar constraint satisfaction problem: it is, after all, an interaction between two protein complexes that requires establishing bonds and avoiding steric clashes. For example, a combination of constraints placed on peptides binding to the a3a TCR [27, 28] was previously identified that explains its apparent promiscuity from a structural biology perspective [29].

As our previous examples of the Hamming distance already show, not all constraint satisfaction problems generate rugged energy landscapes: the key ingredient is *frustration* resulting from constraints that compete with each other. If we extend classic receptor‐antigen interaction models to represent receptors not by just one, but by *multiple* strings—representing different, possibly conflicting constraints that binding antigens need to fulfill—then we obtain such frustration effects in immunogenicity landscapes. Indeed, such multi‐constraint versions of receptor‐antigen interaction models have already been used in the field of artificial immune systems. Over the past decade, work by our group has characterized the computational difficulty of multiple such problems [30, 31, 32, 33, 34]. Our results have shown that there is an important distinction here between contiguous and Hamming distance: while the multi‐constraint contiguous distance remains computationally “easy”, the multi‐constraint Hamming distance is “computationally hard” [35]. We now discuss the implications of these findings for the topology of predicted immunogenicity landscapes from such a model.

**FIGURE 4:**
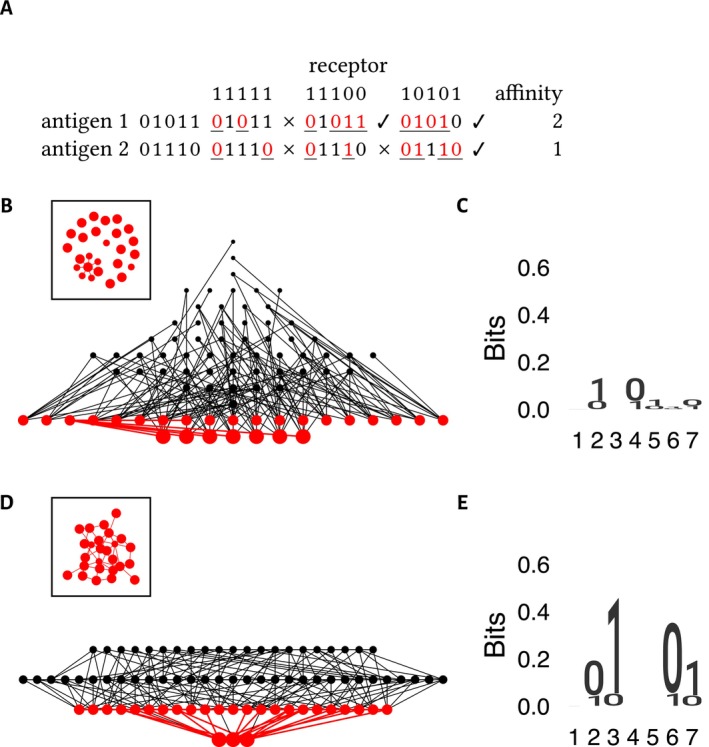
A constraint‐satisfaction model of receptor‐antigen binding can generate rugged immunogenicity landscapes. (A) Illustration of the model. In this example, the receptor consists of 3 strings, and the affinity of an epitope is defined as the number of strings that it has at least 4 complementary bits to (checkmarks). (B,C) Immunogenicity landscape for a receptor consisting of 18 constraints of length 7, giving rise to a rugged landscape. (B) Neighborhood structure graph of the most immunogenic epitopes (as in Figure 3C); inset: Subgraph when only the most immunogenic epitopes (red in the full graph) but not their neighbors are considered, showing that changing a single epitope position would destroy immunogenicity in most cases. (C) Sequence logo of the immunogenic epitopes, showing no strong bias at any position. (D,E) Same as B,C but for a receptor consisting of only 5 constraints, giving rise to a more smooth landscape with a single, connected cluster of immunogenic epitopes (D) that share a recognizable sequence motif (E).

In the multi‐constraint version of the Hamming model, we represent each antigen by a single string as before, but each TCR by multiple strings (Figure 4A). The affinity between a given TCR‐antigen pair is the number of TCR‐strings that have at least k complementary positions with the antigen. In previous work [32], we proved that even finding a single matching antigen for a given TCR is as hard as many other known hard problems in computer science [36]. Therefore, for constraints that are difficult to satisfy simultaneously, very complex immunogenicity landscapes can be generated where single mutations can lead to large changes in immunogenicity (Figure 4B). There can then be many high‐affinity strings that are not similar to each other (Figure 4B, inset) and do not share recognizable sequence motifs (Figure 4C).

However, not all immunogenicity landscapes generated by the constraint satisfaction model of TCR‐pMHC binding are this rugged. Suppose the number of constraints is small or the constraints are easy to satisfy simultaneously. In that case, we can still get smooth landscapes where there is broad sequence similarity between all immunogenic antigens (Figure 4D,E). In summary, if we take the view that TCR‐pMHC binding is essentially a constraint satisfaction problem, then the immunogenicity landscape could be convex or non‐convex, monospecific or multispecific, depending on the number and type of the constraints.

In summary, we have so far argued that classic theoretical models of receptor‐antigen interaction would predict that TCR‐pMHC immunogenicity landscapes should be monospecific, though not necessarily convex. However, straightforward extensions of such models, which would be in line with the view of TCR‐pMHC binding as a process that requires fulfilling multiple, independent constraints, predict that these landscapes may well feature non‐convex, multispecific recognition regions. We will now examine published data for possible clues as to which of these predictions could be closer to reality.

## 5 Mutation Matrix Data Suggest Non‐Convex Recognition Regions

Due to the vast number of possible TCR‐pMHC combinations, much of the TCR‐pMHC shape space will remain unexplored in the foreseeable future. However, several large mutagenesis studies were performed that allow us to explore the immunogenicity landscape. Here, we will consider two systematic investigations of TCR‐pMHC interaction where a large number of peptides were tested against one or several chosen TCRs. The first dataset, already visualized in Figure 2, provides important information on the effect of single point mutations on recognition status for a fixed “panel” of TCRs [13, 14].

**FIGURE 5:**
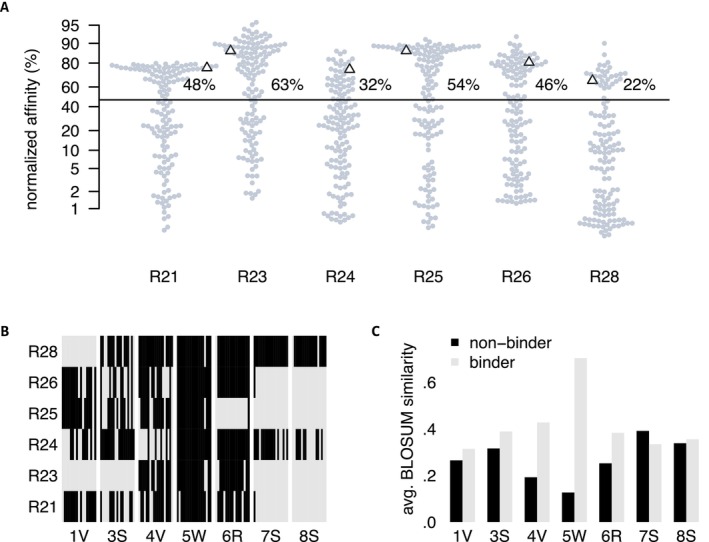
Effect of point mutations on binding affinity for six TCRs that recognize the human cancer neo‐epitope VPSVWRSSL [14] (Figure 2A). (A) Starting from the original neo‐epitope (triangle), each position (except for the MHC anchor positions) was mutated, and the resulting binding affinity was measured (blue points). The horizontal line shows the threshold of ~47% used by Drost et al. to distinguish binders from non‐binders [14]. For clarity, the Y‐axis is transformed to log odds. (B) Binding status after each mutation (vertical stripes, grouped by position in the peptide) for each TCR; dark: Non‐binder, light: Binder. (C) For each position, a similarity measure based on a BLOSUM matrix (see Methods) was averaged over mutations that break immunogenicity (black) versus those that do not (bright). For example, at position 5 (tryptophan), five out of six mutations that retain immunogenicity are to the amino acids tyrosine (similarity score to tryptophan: 0.88) or phenylalanine (0.73), which are both aromatic amino acids like tryptophan.

Strikingly, the data show that a single point mutation is enough to reach a large range of different TCR affinities, with about half of the mutations breaking recognition (Figure 5A). In this aspect, the recognition region of the TCR appears to be very nonconvex. However, some predictability is also seen—for instance, the source peptide shown in Figure 5 contains a tryptophan (W) in position 5, and mutation of this position breaks recognition in the vast majority of cases (Figure 5B). Previous work has indeed argued that (a) middle positions are more important determinants of recognition than peripheral positions, and (b) the presence of tryptophan is associated with better TCR recognition [37]. However, at the other positions, the picture is far more mixed. For example, there are no major differences in amino acid similarity (as measured by the BLOSUM62 substitution matrix) between those mutations that do break immunogenicity and those that do not (Figure 5C).

Overall, the point mutation data show that, if we use sequence similarity as a measure of similarity between peptides, a peptide that is “close” to a known immunogenic one could be similarly immunogenic in many cases but also be much less immunogenic. There is likely no simple, interpretable rule that would allow us to make this distinction—even though it could be possible to identify a few cases in which recognition will definitely be broken, such as tryptophan in the middle position.

## 6 Deep Mutational Screening Data Suggest Multispecific Recognition

To study the extent of TCR cross‐reactivity, Birnbaum et al. conducted a study using affinity‐based selection [38]. They performed a mutational screening of a previously known antigen and selected the sequence for binding affinity to a certain TCR over four selection rounds. They found that over the different rounds, there was a strong preference for one amino acid sequence closely related to the original peptide, with similar sequences also being preferentially selected. Birnbaum et al. concluded that TCR cross‐reactivity is not driven by recognition of entirely unrelated peptides, but by the TCR's tolerance of certain amino acid substitutions within a preferred sequence. While the presence of a single binding motif may suggest a monospecific shape space, this binding motif does not fully represent the entire shape space.

**FIGURE 6:**
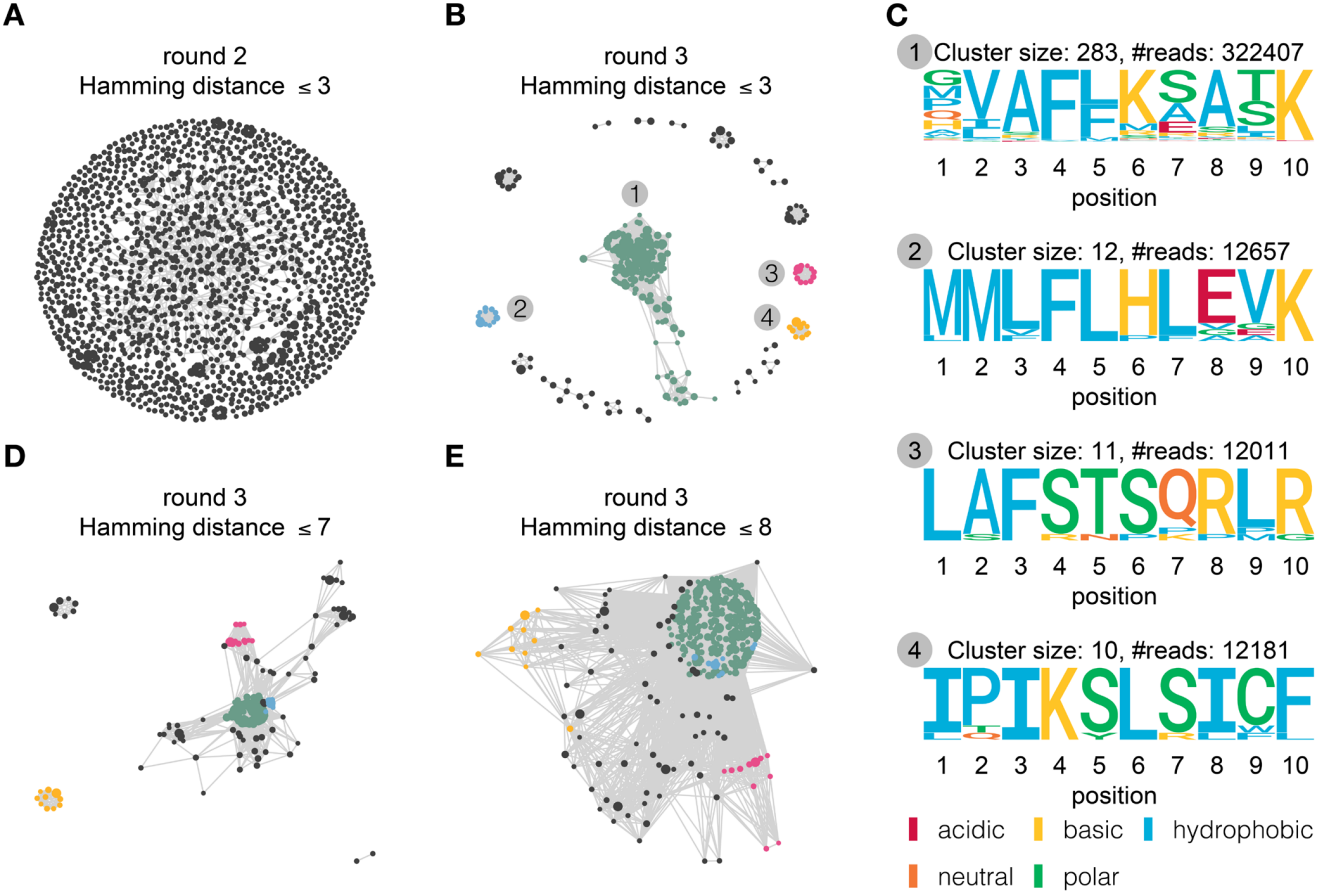
Clustering of affinity‐based selected epitopes reveals distinct and dissimilar groups. Reanalysis of data from Birnbaum et al. [38]. (A) Clustering of epitopes based on a Hamming distance threshold of ≤ 3 after round 2 of affinity‐based selection for the 2B4 TCR. Point sizes represent the number of reads, with only sequences having a read count greater than 5 shown. (B) Clustering of epitopes after round 3 with a Hamming distance threshold of ≤ 3. Point sizes represent the number of reads; only sequences with a read count greater than 5 are shown. (C) Sequence logos for the four largest clusters from (B), where the height of each amino acid corresponds to its probability of occurrence at that position. (D, E) Clustering of epitopes after round 3, with Hamming distance thresholds of ≤ 7 (D) and ≤ 8 (E). Point sizes represent the number of reads, with only sequences having a read count greater than 5 shown.

Indeed, in contrast to the predominant selection of similar sequences, a re‐analysis of the data reveals additional complexity in the recognition profile. Clustering analysis of the selected epitopes for a single TCR (2B4) shows that, while most sequences are closely related to the sequence that selection converges toward, there are also clusters of epitopes that differ significantly from this consensus sequence. Initially, after round 2, the dataset still contained a diverse array of sequences when clustered with a maximum Hamming distance of 3 between the sequences (Figure 6A). However, by round 3, more defined clusters of related sequences became evident, with previously observed noise diminishing (Figure 6B). This clustering analysis reveals the presence of distinct groups of epitopes (Figure 6C), including some clusters with a Hamming distance of 7 between each pair of sequences (Figure 6D), where no clear positional preference for specific amino acids is observed. Only after applying a Hamming distance threshold of 8 were all sequences clustered into a single group (Figure 6E).

Hence, the data by Birnbaum et al. [38] do support the notion of multispecific recognition: the studied TCR cross‐reacts with a diverse range of epitopes with very little similarity between them. This suggests that the global structure of the shape space for a single TCR may be driven not only by tolerance for substitutions in certain positions but also by a broader range of sequence diversity.

## 7 Discussion

The TCR‐pMHC interaction is a complex process that involves two heterodimers and a peptide. Even modern AI tools such as AlphaFold are, therefore, not yet able to accurately model TCR‐pMHC interactions [39, 40]. Since sequence data is much more easily available than structural information, there have been many efforts to predict functional aspects of the TCR‐pMHC interaction, such as its affinity, directly from sequence data. However, these efforts have so far been limited in their success [41, 42]. Similarly, it has been challenging to identify clear signs of repertoire‐shaping processes such as positive [43] and negative [44] selection in TCR sequence datasets. Here, we revisited one of the oldest surviving paradigms in theoretical immunology—the idea of shape space—in search of an intuitive explanation of why these problems might be so hard.

We have seen that if the affinity of TCR‐pMHC interactions imposes conflicting constraints on the peptide sequence—which we think is not an entirely unreasonable idea [29]—then the important assumption of a smooth and monospecific immunogenicity landscape, which underlies existing sequence‐based predictions of immunogenicity either implicitly [37] or explicitly [45], may not fully hold. By re‐analyzing data from three important larger‐scale studies of TCR‐pMHC interactions, we found evidence that there could be at least some degree of smoothness in the pMHC immunogenicity landscape—otherwise, it would be hard to explain why so many point mutations of immunogenic T cell epitopes are still immunogenic. On the other hand, we also found evidence that peptides with seemingly unrelated sequences can be recognized by the same TCR, possibly representing multiple different binding configurations.

Because full TCR‐pMHC binding prediction has remained elusive, researchers have instead tried to identify generic features of pMHCs or TCRs that correlate with interaction strength. For example, Calis et al. [37] showed that the presence of large and aromatic residues in MHC‐I presented peptides is associated with higher immunogenicity, and Frankild et al. argued that peptides with sequences similar to those present in human self are less likely to be immunogenic [45]. On the TCR side, we have found that hydrophobic amino acids contribute to higher reactivity against self peptides [43] and others have found the same amino acids to be enriched in highly self‐reactive regulatory T cells [46, 47]. While such features become visible when investigating large sequence datasets, they generally explain only a small fraction of immunogenicity. Indeed, a non‐convex, multispecific shape space topology would severely restrict how much we can do with simple, interpretable TCR or pMHC features.

Predicting the effect of point mutations is an especially important problem in neoantigen‐based cancer immunotherapy. Currently, hundreds of candidate peptides may need to be screened to find one that produces a robust immune response [48, 49]. Additionally, misprediction of TCR target peptides is a serious risk as this can lead to fatal adverse events [27, 28]. A further complicating factor is that the T cell repertoire and MHC background are very different between people, meaning that immunogenicity predictions may even have to be personalized to be useful. Such fully personalized predictions would, however, require full information on an individual's TCR repertoire, which remains impossible to obtain for the time being—even with the most advanced sequencing technology, we can only sample a tiny fraction of an individual's T cells. Hence, for immunotherapy applications, the problems posed by a multispecific and non‐convex T cell epitope landscape would be exacerbated even further.

All this seems unsatisfying, but things could be worse: For protein folding, models have predicted a *glassy* energy landscape characterized by a huge number of local minima [50], but still, most natural proteins do fold into stable structures, perhaps as a result of natural selection favoring funnel‐like energy landscapes [11]. Compared to protein folding, the number of constraints a pMHC needs to satisfy to be recognized by a given TCR may well be lower. Our re‐analysis of the data by Birnbaum et al. [38], limited as it may be, suggests a landscape more accurately described as *oligospecific* rather than multispecific.

Overall, we suggest that the decades‐old concept of shape space, updated for T cells in light of recent data, is a useful theoretical device to grasp, model, and perhaps ultimately help solve many of the current difficulties we face with understanding and predicting immunogenicity.

## 8 Methods

### 8.1 Ethics

No new data was collected for preparing this manuscript. The re‐analyzed data was generated in vitro and did not involve human subjects.

### 8.2 Re‐Analysis of TCR‐pMHC Affinity Data

To demonstrate the concept of shape space for TCR‐pMHC interactions, we embed TCRs and peptides in 2D space based on their binding data in a process called *multidimensional unfolding*. Unfolding of the TCR‐pMHC peptide binding data as published by Drost et al. [14] was performed using the R package “smacof”, version 2.1.7 [51]. Ordinal unfolding was used (which operates on the ranks of the distances [52]), as had been suggested by Lapedes et al. [6], to account for possible non‐metric structure of the shape space.

To estimate the extent to which a given point mutation would change the peptide's position in shape space, we used the BLOSUM62 substitution matrix [53] as follows. The matrix entries are log odds (ranging from −4 to 11) of finding one amino acid in exchange for another. The entries were projected to the interval $\left[0,1\right]$ for easier interpretation by applying the inverse logit transformation $e^{x}/\left(1+e^{x}\right)$. For example, a BLOSUM score of 0 (e.g., alanine versus cysteine) is mapped to 0.5, and a score of −4 (cysteine versus glutamic acid) is mapped to 0.018.

### 8.3 Re‐Analysis of Peptide Screening Data

We used data from Birnbaum et al. [38] to analyze the affinity‐based selection of epitopes for the 2B4 TCR. We calculated the Hamming distance between the epitope sequences to perform a clustering analysis of this selection data. Sequences containing ambiguous amino acids (denoted by asterisks) or with a read count of fewer than 5 were excluded from the analysis. We generated sequence logos for the largest clusters using the ggseqlogo R package [54].

### 8.4 Constraint‐Based TCR‐pMHC Interaction Model

We used the farthest string problem (which is computationally equivalent to the related *closest string problem*) [36, 55] to model the constraints imposed by a TCR on its matching pMHC. Here, we give a brief formal definition of the problem for the sake of reproducibility. In the farthest string problem, we are given n strings $r_{1},\ldots ,r_{n}$ of the same length ℓ over an alphabet Σ (we use the binary alphabet $\Sigma =\left\{0,1\right\}$), a threshold k≤ℓ, and another string a. We then determine the number of strings $r_{i}$ for which $d\left(a,r_{i}\right)\geq k$, where d denotes the Hamming distance. This problem is NP‐hard, i.e., it is hard in the sense that no algorithm is known that can determine, in time polynomial with respect to n and ℓ, whether for given strings $r_{1},\ldots ,r_{n}$ and threshold k there exists a string a such that $d\left(a,r_{i}\right)\geq k$ holds for all $r_{i}$. Further, if some such algorithm were found, then this algorithm would immediately also solve a multitude of other similarly hard problems in polynomial time, although no polynomial time algorithm is currently known to solve any of them.

## Author Contributions

F.B., G.S. and J.T. designed the research. F.B. and J.T. analyzed the T.C.R.‐pMHC interaction datasets and visualized the results. G.S. and J.T. designed and analyzed the constraint‐based shape space model. G.S. and J.T. performed simulations of the multi‐constraint shape space models. F.B. and J.T. created the figures shown in this paper with input from G.S. F.B., G.S. and J.T. drafted the manuscript.

## Funding

This work was supported by Nederlandse Organisatie voor Wetenschappelijk Onderzoek, (VI.Vidi.194.084). ZonMw, (10710062310009).

## Conflicts of Interest

The authors declare no conflicts of interest.

## Data Availability

Data sharing not applicable to this article as no new datasets were generated during the current study. Code Availability Statement: Code used to perform the simulations and data analyses shown in this manuscript is available on Zenodo [56].
